# Practice Recommendations for Reducing Anticholinergic Burden in People with Intellectual Disability and Mental Health Difficulties

**DOI:** 10.1192/bjo.2025.10437

**Published:** 2025-06-20

**Authors:** Reena Tharian, Sreeja Sahadevan, Melissa Hicks, Elizabeth Patteril, Regi Alexander

**Affiliations:** 1 Hertfordshire Partnership University NHS Foundation Trust, Norwich, United Kingdom; 2 Norfolk and Suffolk Foundation NHS Trust, Norwich, United Kingdom

## Abstract

**Aims:** In people with intellectual disability (ID), anticholinergic burden (ACB) is associated with multimorbidity, polypharmacy, and premature mortality. A previously reported baseline audit evaluated ACB over a period of 3 months in two inpatient units. This project reports on the development of practice recommendations and audit standards based on that work.

**Methods:** The baseline audit results were discussed in two peer group meetings of prescribers and two multidisciplinary continuing professional development (CPD) sessions. Based on a qualitative analysis of themes from these discussions, good practice recommendations and an Anticholinergic Quick Checklist (ACQC) for screening were finalized.

**Results:** The practice recommendations were

1. The indication(s) and rationale for prescribing all psychotropic medications, including those with anticholinergic properties, should be clearly stated.

2. Consent-to-treatment procedures or best-interest decision-making processes should be followed and documented.

3. ACB of the patient’s medication regime should be calculated using an instrument like the AEC scale (Medichec), ACB calculator, or equivalent.

4. Side effects of psychotropic medication and treatment outcomes should be monitored using standardised scales like the LUNSERS, GASS, CGI Efficacy Index, CGI Global Improvement, or equivalents.

5. There should be regular monitoring of treatment response and side effects of all prescribed psychotropic medications, including those with anticholinergic properties.

6. There should be regular review and evaluation of the need for continuation or discontinuation of all prescribed psychotropic medications, including those with anticholinergic properties.

**Conclusion:** There is a need to evaluate the psychometric properties of the Anticholinergic Quick Checklist (ACQC). Larger scale studies and service evaluations are needed to further improve clinical practice in addressing anticholinergic burden (ACB) in people with intellectual disabilities (ID).

